# Cardiovascular, electrophysiologic, and hematologic effects of omega-3 fatty acids beyond reducing hypertriglyceridemia: as it pertains to the recently published REDUCE-IT trial

**DOI:** 10.1186/s12933-019-0887-0

**Published:** 2019-06-24

**Authors:** Omar Sheikh, Anthony G. Vande Hei, Ayman Battisha, Tarek Hammad, Son Pham, Robert Chilton

**Affiliations:** 1 Cardiology Division, University Texas Health Science Center at San Antonio, 7403 Wurzbach Road, San Antonio, TX 78229 USA; 2 Cardiology Division, Brooke Army Medical Center, San Antonio, TX USA; 3 Cardiology Division, University of Massachusetts Medical School-Baystate, Springfield, MA USA

**Keywords:** Omega-3 polyunsaturated fatty acids, Hypertriglyceridemia, Hyperlipidemia, Diabetes mellitus

## Abstract

Heart disease continues to affect health outcomes globally, accounting for a quarter of all deaths in the United States. Despite the improvement in the development and implementation of guideline-directed medical therapy, the risk of adverse cardiac events remains substantially high. Historically, it has been debated whether omega-3 polyunsaturated fatty acids provide clinical benefit in cardiac disease. The recently published REDUCE-IT trial demonstrated a statistically significant absolute risk reduction of 4.8% in its primary endpoint (cardiovascular death, nonfatal myocardial infarction, nonfatal stroke, coronary revascularization, or unstable angina) with the use of icosapent ethyl, which is a highly purified eicosapentaenoic acid (EPA) ethyl ester. However, the mechanism of action of omega-3 fatty acids is not commonly discussed. Moreover, the use of EPA was not without risk, as the incidence of atrial fibrillation was increased along with a trend towards increased bleeding risk. Thus, our aim is to help explain the function of purified EPA ethyl ester, especially at the molecular level, which will ultimately lead to a better understanding of their clinically observable effects.

## Background

The decline in coronary artery disease (CAD)-related mortality over the last four to five decades was regarded as a major breakthrough [1]; however, multiple rebutting reports from recent years that demonstrate an increasing trend towards higher mortality rates from CAD in specified patient populations exist [2, 3]. Despite advancements in prevention and treatment strategies, current data illustrate that heart disease accounts for a quarter of all deaths in the United States.

Omega-3 fatty acids are not commonly utilized in the clinical setting because of the lack of uniform evidence of their ability to reduce the incidence of adverse cardiovascular events. The Cochrane group reported similar findings, demonstrating no significant benefit [4]. The lack of evidence has led physicians away from recommending omega-3 fatty acids and towards dietary modifications, exercise, and weight loss. By contrast, Yokoyama et al. [5] reported that eicosapentaenoic acid (EPA) significantly reduces major adverse cardiovascular events by 19% when used for secondary prevention of cardiovascular disease. The recently published landmark REDUCE-IT trial [6] examined the use of EPA in addition to statin therapy in patients with hypertriglyceridemia to further explore its role in the appropriate clinical setting.

Multiple factors associated with omega-3 fatty acids and inconsistent results of published studies have been reported, which include, but are not limited to, dosage, preparation, studied population, clinical setting (primary vs. secondary prevention), and background therapy [7]. Furthermore, their cellular mechanism of action and how they produce their clinical effects are not commonly discussed. Thus, in this discussion, we aimed to review the composition of omega-3 polyunsaturated fatty acids, their multiple pharmaco–physiologic pathways, and their resultant effects on the cardiovascular system.

## Overview of the REDUCE-IT trial

Utilizing 473 participating sites across a total of 11 countries, Bhatt et al. [6] randomized patients into two groups: the intervention group, which received 2 g of icosapent ethyl twice daily and the placebo group. The inclusion criteria were as follows: patients aged ≥ 45 years with known cardiovascular disease, those aged ≥ 50 years with diabetes mellitus and at least one additional risk factor, and those with a fasting triglyceride level of 135–499 mg/dL and a low-density lipoprotein cholesterol (LDL-C) level of 41–100 mg/dL. However, an amendment to the inclusion criteria was made in 2013; the lower limit of acceptable triglyceride level increased from 135 mg/dL to 200 mg/dL. A total of 8179 patients underwent randomization, and 70.7% of whom were enrolled on the basis of secondary prevention. Between the two treatment arms, the distribution of patients with diabetes was 58%, and the median triglyceride level was 216 mg/dL.

Patients were followed for an average of 4.9 years; the median change in triglyceride level from baseline to 1 year was a decrease of 18.3% in the icosapent ethyl group and an increase of 2.2% in the placebo group. The median LDL-C level increased from baseline by 3.1% in the icosapent ethyl group and by 10.2% in the placebo group.

The primary endpoint of the study was a composite of cardiovascular death, nonfatal myocardial infarction (MI), nonfatal stroke, coronary revascularization, or unstable angina. Secondary endpoints included cardiovascular death, nonfatal MI, or nonfatal stroke. The primary outcome of interest occurred in 17.2% of patients in the icosapent ethyl group and in 22.0% in the placebo group (p < 0.001), with a number needed to treat (NNT) of 21 over a median of 4.9 years. Secondary endpoints were observed in 11.2% of patients in the icosapent ethyl group and in 14.8% in the placebo group (p < 0.001). With regard to adverse events, the rate of atrial fibrillation (AF) was statistically higher in the icosapent ethyl group (5.3%) than in the placebo group (3.9%), as was peripheral edema (6.5% vs. 5.0%), with a trend toward increased rate of serious bleeding (2.7% vs. 2.1%; p = 0.06) in the intervention group.

## Basic science of omega-3 fatty acids

### Biochemistry

Molecular and interventional studies have demonstrated the benefits of omega-3 fatty acids in the primary and secondary prevention of cardiovascular disease. These benefits arise from multiple, complex interactions, including inflammatory mediators, lipid metabolism, alteration of cardiac ion channels, cell membrane chemistry and physiology, down-stream cell signaling pathways, and anti-thrombotic pathways, to name a few. To gain further understanding, it is critical to understand the basic science regarding omega-3 fatty acids.

Omega-6 and omega-3 fatty acids are polyunsaturated fatty acids (PUFA) that represent the two classes of essential fatty acids, and they must be derived from diet as humans cannot synthesize them in sufficient quantities. The difference between them is based on the location of the fatty acid’s double bond from the methyl end. Omega-6 fatty acids, most notably arachidonic acid (AA), have their first double bond between the fifth and sixth carbons, and omega-3 fatty acids, such as EPA and docosahexaenoic acid (DHA), have their first double bond between the third and fourth carbons.

Omega-3 fatty acids are derived from a-linoleic acid (ALA), which is found in plant oils and marine oils, and omega-6 fatty acids from linoleic acid, which is commonly found in vegetable oils. Once ALA is consumed, it is metabolized by a desaturase, elongase, and another desaturase enzyme, which results in EPA formation. EPA undergoes further enzymatic action and eventual peroxisomal β-oxidation to yield DHA [8, 9]. The EPA derivative has specifically shown promise in the REDUCE-IT trial.

Through enzymatic conversion, PUFA could form 20 carbon atom molecules called eicosanoids (eicosa = 20). Specifically, eicosanoids are produced from membrane phospholipids by the action of phospholipases and could form prostaglandin(s)/thromboxane via cyclooxygenase (COX1/2) and leukotrienes/lipoxins via lipoxygenase. AA (omega-6) usually produces pro-inflammatory eicosanoids, named two-series prostanoids (prostaglandin E_2_, PGD_2_, PGI_2_ thromboxane A_2_) and four-series leukotrienes (LTB_4_, LTE_4_). These pro-inflammatory mediators produce a wide array of actions, such as vasodilatation, vasoconstriction, leukocyte activation, platelet aggregation, and production of reactive oxygen species. PGE_2_ specifically causes fever and increases vascular permeability to allow for chemotaxis of leukocytes and the formation of edema [10]. By contrast, EPA-derived eicosanoids are generally anti-inflammatory, including three-series prostanoids and five-series leukotrienes (the series dictates where the double bond lies) [11–13]. EPA has a variety of mechanisms to produce their anti-inflammatory effect, including the regulation of transcription factors that are responsible for the inflammatory cascade, such as NF-κB, which regulates the transcription of genes for inflammatory cytokines, i.e., Il-1β. Moreover, EPA competes with AA for cell membrane phospholipid synthesis, with preferential splitting of EPA by phospholipase A_2_ instead of AA, which in turn would yield EPA-derived eicosanoids, thereby favoring the production of anti-inflammatory eicosanoids instead of the pro-inflammatory AA-derived eicosanoids [9].

The ratio of EPA/AA has been found to correlate with cardiovascular risk and atherosclerotic progression. A lower serum EPA/AA ratio is associated with an increased risk of cardiovascular disease and coronary events [14, 15]. In the Japan EPA Lipid Intervention Study, a significant decrease in cardiac risk in patients with an EPA/AA ratio of > 0.75 was found [16]. Interestingly, the study also found that increased EPA, but not DHA, levels are associated with decreased major coronary events.

Two families of proteins derived from EPA and DHA called protectins and resolvins were discovered to play a pivotal role in resolving inflammation [17, 18]. Resolvins have two structural forms: resolvin E and D. Resolvin E is formed by the action of 5-lipoxygenase on EPA-derived mediators and inhibits neutrophil chemotaxis, specifically transendothelial migration. Protectins and resolvins derived from DHA both play a role in blocking t-cell and neutrophil migration, respectively.

**Fig. 1:**
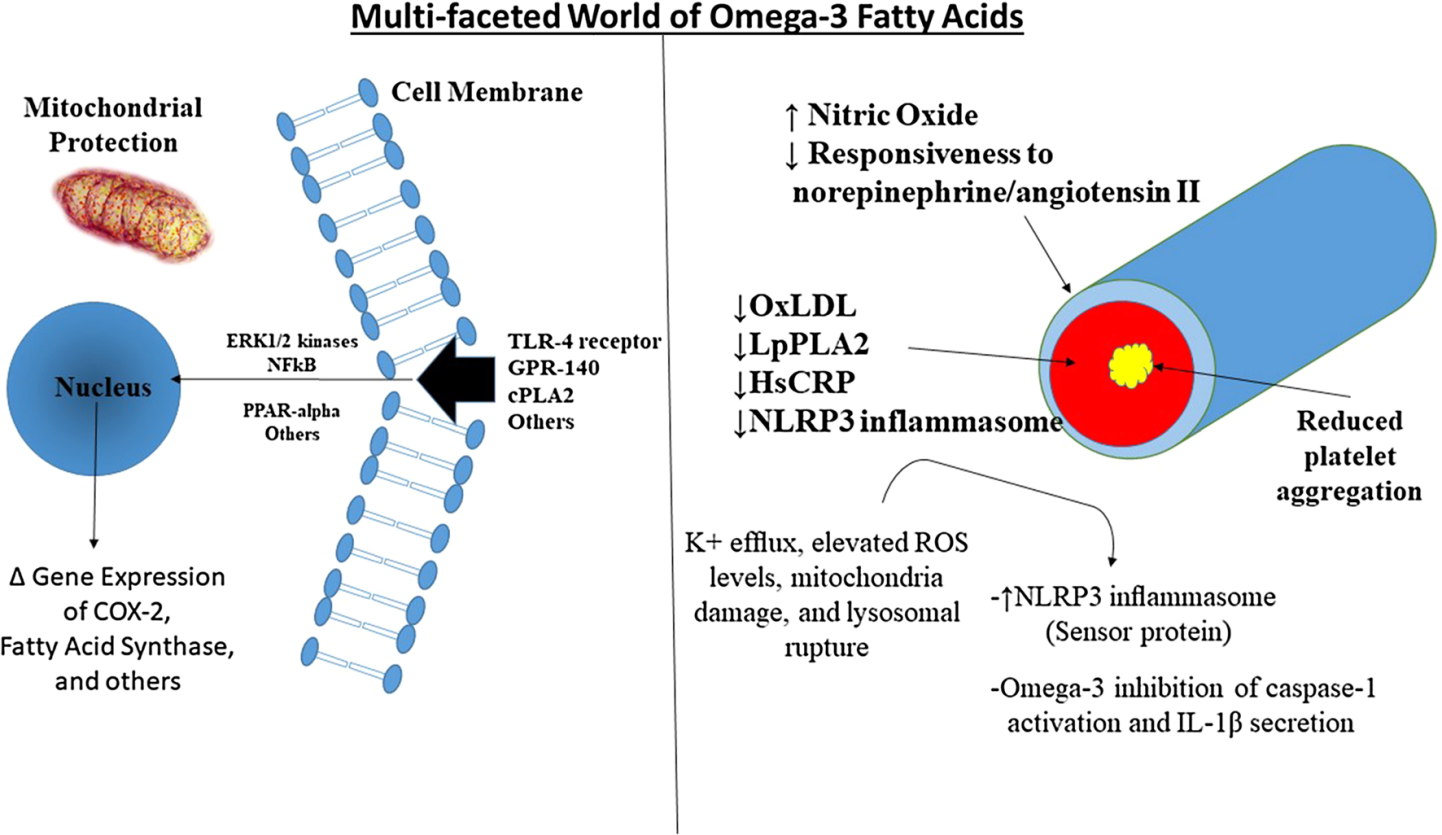
Multi-Faceted World of Omega-3 Fatty Acids. 1) Improved endothelial function seen with omega 3 fatty acids [102] in addition to reduced response to norepinephrine and angiotensin II. Moreover, there is limited evidence available that there is improvement in left ventricular function [103]. 2) Improvement in inflammation noted by a reduction in markers of inflammation including oxidized LDL (ox-LDL), Lipoprotein-associated phospholipase A2 (LpPLA2) and High-sensitivity C-reactive protein (hsCRP) seen in the ANCHOR Trial [104]. 3) Reduction in platelet aggregation has been reported in a number of small studies [59]. 4) Omega-3 fatty acids have shown to prevent inflammation and metabolic disorder through inhibition of inflammasome activation [105]. *TLR4* toll-like receptor 4, *GPR-140* G-protein coupled receptor, *cPLA2* cytosolic phospholipase 2 ERK1/2 = protein-serine/threonine kinases involved in the ras-raf-mek-erk pathway transduction cascade involved in gene transcription NLRP3 Inflammasome = signal complex which activate inflammatory caspaces and IL-β to upregulate inflammation. *ROS* reactive oxygen species

PUFAs can influence gene transcription by binding to nuclear receptors, such as peroxisome proliferator-activated receptor (PPAR). PPARs are ligand-activated transcriptors that regulate lipid and carbohydrate metabolism, cellular proliferation, and inflammation (Fig. 1) [12, 19]. They are the first identified transcription factors that are regulated by fatty acids. Three PPAR isoforms are PPAR-α, PPAR-β/δ, and PPAR-γ. PPAR-α is mostly involved in fatty acid metabolism, including β-oxidation of fatty acids, decreased hepatic triglyceride secretion, increased lipoprotein lipase activity with very low-density lipoprotein (VLDL) clearance, and increased production of high-density lipoprotein cholesterol, thereby promoting a favorable hypo-lipemic effect [19, 20]. Anti-diabetic agents known as fibrates have been designed to specifically target PPAR-α. PPAR-γ modulates fatty acid storage and glucose metabolism; specifically, they reduce insulin resistance, thereby stimulating lipid uptake and adipogenesis. The anti-diabetic thiazolidinediones (glitazones) target the PPAR-γ receptor [20]. PPAR-β/δ is mostly expressed in the intestinal tract and maintains homeostasis for cell turnover and inflammation. Grimaldi et al. reported that PPAR-β/δ could promote the breakdown of fatty acids in the skeletal muscle and has an insulin sensitizer effect, thus making it a possible future treatment target for diabetes [20, 21].

Most studies, including meta-analysis and randomized controlled trials (RCT), have used various combinations of EPA and DHA, accounting for the isolated effects between EPA and DHA is difficult. Nevertheless, they are believed to have specific actions depending on the particular part of the body [11]. EPA’s lipophilic nature allows it to bind to the phospholipid bilayer in blood vessels where it could modulate inflammation and endothelial dysfunction and inhibit lipid oxidation at sites of plaque formation. EPA has shown to improve HDL function in patients with coronary artery disease by promoting cholesterol efflux, neutralize free radicals, and provide anti-inflammatory effects [22, 23]. DHA’s effects are largely restricted to the nervous system; most of their effects are involved in the neuronal or retinal membranes. Accordingly, a previous study reported that dietary DHA is needed to provide maturation of the retina and visual cortex and could restrict cognitive decline with aging [24].

Allaire et al. [25] performed a randomized, double-blind, randomized controlled trial (RCT) comparing EPA and DHA head-to-head in 106 women and 48 men. Their primary endpoint was the effect on inflammatory markers, and secondary outcomes included the effect on lipid levels. They reported that DHA significantly increases adiponectin and HDL compared to EPA, and DHA showed a greater reduction in triglycerides compared to EPA. Moreover, the anti-lipid oxidant effects of EPA appear to be isolated, as supplementation with other triglyceride-reducing agents has not shown to be effective. Mason et al. showed that fibrates, niacin, gemfibrozil, and vitamin E do not significantly affect LDL/VLDL oxidation, and DHA inhibits oxidation over a shorter period of time compared to EPA [26].

Hence, EPA and DHA are believed to have differential effects on cardiometabolic risk factors, although the results are controversial as many of the previous studies are limited by a small sample size. Currently, a large RCT for a head-to-head comparison between EPA and DHA has not been conducted [27].

### Effect on endothelial function

Endothelial dysfunction plays a role in the development of atherosclerosis, and it is characterized by a pro-inflammatory, thrombotic state with reduced vasodilatory capacity. Risk factors for endothelial dysfunction include hypertension, hyperlipidemia, cigarette smoking, and metabolic syndrome. The mechanism by which PUFA improves endothelial function is not completely understood; nevertheless, the most plausible mechanism elucidated includes an increase in nitric oxide synthesis via DHA/EPA-induced activation of endothelial nitric oxide synthase, decrease in free radical production, and suppression of endothelial/vascular activity by decreasing the expression of endothelial adhesion molecules (vascular cell adhesion molecule-1), which leukocytes use to adhere to the vasculature to promote inflammation and monocyte activation [28].

### Antioxidant effect

Patients with hyperlipidemia and type 2 diabetes treated with 1.8 g of EPA daily for 6 months were found to have increased levels of adiponectin, with levels approaching those observed in the non-diabetic control population. Adiponectin was found to suppress monocyte adhesion to the endothelium, decrease NO [29], and reduce the uptake of oxidized LDL, which could play an important role, as increased oxidized LDL has been linked to future cardiovascular events in healthy middle aged men [30]. PUFAs decrease inflammation via protectins and resolvins, which, as previously described, decrease neutrophil and T-cell recruitment and reduce inflammation in atherosclerosis.

### Anti-atherosclerotic benefits

EPA has shown to increase fibrous cap thickness, which helps stabilize and prevent atherosclerotic plaque rupture, thereby accounting for its cardioprotective properties. A study by Yamano et al. showed that administration of EPA 1.8 g/day significantly increases fibrous plaque thickness in patients with acute coronary syndrome (ACS) based on optical coherence tomography (OCT). Serial OCT was performed on a non-culprit plaque with a percent diameter stenosis of 30% to 70% in the non-culprit vessel. The control group also had increased fibrous plaque thickness; however, the relative change in fibrous plaque thickness was higher in the EPA group than in the control group (131 ± 35% vs. 106 ± 15%; p = 0.001) [31]. Similarly, EPA 1.8 g/day for 6 months plus statin therapy has shown to decrease lipid plaque volume (p = 0.007) and increase fibrous plaque volume (p = 0.01) as measured by intravascular ultrasound [32]. As previously mentioned, a lower serum EPA/AA is associated with cardiovascular events; moreover, based on OCT imaging, it is also associated with increased coronary plaque vulnerability in patients with ACS, and the EPA/AA ratio showed a significant positive correlation with fibrous cap thickness [33].

## Diabetes, lipids, and icosapent ethyl (eicosapentaenoic acid ethyl ester)

Omega-3 fatty acids have been considered for the treatment of hypertriglyceridemia, especially in diabetic patients. The leading cause of morbidity and mortality in patients with diabetes is atherosclerotic cardiovascular disease (ASCVD) [34]. The exact mechanisms that link ASCVD to diabetes remains unknown [35]. Nonetheless, several mechanisms/pathophysiological pathways have been explored, including oxidative stress, dyslipidemia, endothelial dysfunction, vascular calcification, hypercoagulable state, and insulin resistance [36]. PUFA could modify a number of these pathophysiological mechanisms. Thus, a recent science advisory comment from the American Heart Association in 2018, which is derived from observational, experimental, and RCTs, recommended 1–2 seafood meals per week (which would provide 250 mg of EPA and DHA per week) to reduce the risk of congestive heart failure, coronary artery disease/ischemic heart disease, and sudden cardiac death [37]. However, in 2017, AHA released a statement reporting that fish oil is appropriate for secondary prevention of CAD in patients with diabetes, but not for primary prevention [7]. Until recently, the use of PUFAs has been associated with conflicting reviews.

A study by Poreba et al. [35] investigated the association between glycemic control in 74 patients with type 2 diabetes and ASCVD, specifically in relation to the differences in the levels of omega-3 PUFA and omega-6 PUFA and inflammatory status. They found that patients with HbA1c ≥ 7.0% (poor glycemic control) have decreased levels of EPA and total omega-3 PUFA, lower EPA/AA ratio in their serum phospholipid fraction, higher omega-6/omega 3 ratio, and increased high-sensitivity C-reactive protein (hsCRP) concentrations. Serum phospholipid fraction indicates the dietary fatty acid intake of an individual over the past few weeks and their endogenous fatty acid metabolism. These findings were similar to the study by Takahashi et al. which demonstrated that patients with type 2 diabetes and a history of MI have lower omega-3 PUFA level and EPA/AA and DHA/AA ratios [38].

From the REDUCE-IT trial and similar studies, EPA ethyl ester has been the most vigorously studied compound to date, as its use could lower serum triglyceride levels by 20–50% [39]. In addition, it could also reduce the rate of hepatic production of VLDL and possibly increase hepatic clearance of VLDL [40, 41]. The three primary mechanisms associated with the reduction of VLDL include fatty acid beta-oxidation, with reduced delivery of non-esterified fatty acids to the liver or a reduction in fatty acid synthesis; consumption by increasing phospholipid synthesis; and reduced activity of triglyceride-synthesizing enzymes [42]. Further evidence of EPA’s triglyceride-lowering effect was observed in a subset of the ANCHOR study, which was an efficacy and safety study of 702 patients with elevated triglycerides (200–500 mg/dL) on statin therapy (> 70% of patients had diabetes). They concluded that icosapent ethyl 4 g/day reduces triglycerides by 23%, LDL by 6%, non-HDL by 14%, and VLDL by 28% [43].

In 2018, the A Study of Cardiovascular Events in Diabetes (ASCEND) trial, through a 2 × 2 factorial design, randomized 15,480 patients with diabetes to primary prevention aspirin or placebo, with an average follow-up of 7.4 years. The primary aim of the study was to assess the efficacy and safety of 100 mg of aspirin daily for the prevention of cardiovascular events and cancer in patients with diabetes without known cardiovascular disease. The study also examined whether omega-3 PUFA 1 g/day decreases cardiovascular events compared to a placebo (olive oil capsules). The study reported that omega-3 supplementation did not prevent serious cardiovascular events in patients with diabetes, and aspirin has a modest benefit (1.1% absolute risk reduction, NNT = 91) in the primary prevention of cardiovascular events in well-controlled diabetes and is associated with an increased risk of major bleeding. Of note, the mean HbA1c of 80% of the patients at enrollment was < 8.0, and most patients were on a statin at baseline. Therefore, these results may not be generalizable to all patients with diabetes, especially those with uncontrolled diabetes who are at a higher risk of cardiovascular events. Similarly, the Vitamin D and omega-3 (VITAL) trial, which is a randomized controlled trial, investigated 25,671 individuals from the general population (men, aged ≥ 50 years; women, aged ≥ 55 years); 51% of the patients were women. The study aimed to assess the primary prevention of cancer and cardiovascular disease with 1 g of omega-3 fatty acids (as a fish-oil capsule containing 840 mg of omega-3 fatty acids [460 mg of EPA and 380 mg of DHA]) and 2000 IU of vitamin D3 daily. Primary end points were major cardiovascular events (a composite of MI, stroke, or death from cardiovascular causes) and invasive cancer of any type. The mean follow-up was 5.4 years. The study found no reduction in the incidence of cardiovascular events or cancer compared to placebo. It is important to note that the ASCEND and VITAL trials tested omega-3 fatty acids in patients who did not have previous cardiovascular disease (primary prevention).

In regards to secondary prevention, a meta-analysis of 10 trials by Aung et al. [44] investigated omega-3 fatty acid supplementation for the secondary prevention of cardiovascular disease by randomizing EPA at a dose range of 226–1800 mg/dL in ~ 7000 patients and did not show benefit in patients with a history of cardiovascular disease or in patients at high risk of cardiovascular disease; a meta-analysis by Abelhamid et al. demonstrated similar results [4].

The insignificant results of omega-3 supplementation in the aforementioned trials could be explained by several factors. In the VITAL and ASCEND trials, they administered only 1 g of omega-3 fatty acid and found no benefit; in the REDUCE-IT trial where a 25% decrease in cardiovascular events and mortality was noted in the intervention group, 2 g twice a day (a total of 4 g) was administered (both the placebo and intervention groups had statin therapy). The different doses and durations of omega-3 PUFA could influence the effects of cardiac-metabolic biomarkers [45], which could in turn explain the inconsistent results among the RCT and meta-analyses. Moreover, although the meta-analyses by Aung et al. and Abelhamid et al. found no benefit, a 10% decrease in cardiovascular outcomes with marginal significance was noted. Furthermore, the genetic background of an individual may influence the metabolism and effectiveness of omega-3 fatty acids, as observed among Greenland Inuits [46]. Therefore, further genetic studies in addition to RCT would provide a better understanding of the patients who would most benefit from PUFA [47, 48].

Another study that tested the efficacy and safety of omega-3 fatty acids is the Statin Residual Risk Reduction with EpaNova in High Cardiovascular Risk (STRENGTH) trial, which is an upcoming randomized, double-blind, placebo-controlled study that is expected to be completed by 2020. Similar to the REDUCE-IT trial, this study includes high-risk patients with hypertriglyceridemia and includes men with HDL-C < 42 mg/dL and women with < 47 mg/dL at baseline. The patients are treated with EPA and DHA (4 g/day) [49].

Patients with diabetes are to known to have endothelial dysfunction [50], which is a major risk factor for atherosclerosis [51] and an independent predictor of cardiovascular disease and events [52]. Omega-3 PUFA have been shown to improve endothelial dysfunction, and the plasma level of EPA has been shown to be a marker of vascular function in patients, including those with diabetes with a known endothelial dysfunction [53].

## Hematologic effects of omega-3 fatty acids

Majority of the hematologic effects of PUFA are centered on their antiplatelet effects. As mentioned previously, AA functions as a substrate to produce prostaglandins and thromboxane (TXA_2_), which is a pro-thrombotic, two-series prostanoid produced via the sequential action of phospholipase A_2_, COX-1, and thromboxane synthase. Thromboxane was originally thought to be solely produced and released by platelets; currently, it is known to be produced by other cells as well, including inflammatory and endothelial cells. As the name suggests, it is a potent stimulator of platelet activation, platelet aggregation, and vasoconstriction via smooth muscle contraction, usually after tissue injury or inflammation [54]. It works in an autocrine or paracrine fashion to stimulate and activate adjacent platelets to recruit them to the site of injury. When thromboxane binds to its receptor, platelets undergo a conformational change, resulting in degranulation with release of various vasoactive factors favoring thrombosis and vasoconstriction of the affected vessel [55]. EPA inhibits the activity of thromboxane and thus inhibits platelet activity. The mechanism involved is similar to the anti-inflammatory properties of EPA; if EPA is available in abundant quantities, it will preferentially insert into the phospholipid membrane and compete with AA for the eicosanoid enzymes, leading to less production of thromboxane and more production of the three-series variant of thromboxane, TXA_3_, which is a much weaker platelet stimulant. EPA-induced direct suppressive effect on the thromboxane receptor has also been reported [56].

The effects of omega-3 PUFA on platelet aggregation and thrombosis have long been described; Dyerberg et al. [57] reported in 1978 that Greenlander Eskimos are prone to bleeding because they had higher EPA levels and lower AA levels in their membrane phospholipids. Thus, EPA-enhanced diet could lead to an anti-thrombotic state. More extensive research performed by Gryglewski found reduced platelet aggregation in the presence of EPA [58]. Similarly, in a prospective study by Li and Steiner, normal patients (four women and four men) received 6 g of EPA for 25 days. They found a > 60% decrease in platelet adhesion to fibrinogen and collagen type 1 with fish oil, and platelet pseudopodia was reduced [59]. Platelets are known to strongly adhere to collagen type 1 via their glycoprotein VI receptor during the hemostatic process. As platelets are activated to promote hemostasis, they undergo a shape change, by rearrangement of their cytoskeletal protein, from their regular discoid shape to “spaghetti-like” projections called pseudopodia [60, 61]. Therefore, Li and Steiner’s findings represent an overall reduction in platelet function.

Concerns regarding the theoretical bleeding risk associated with omega-3 fatty acids have been raised, and some would suggest that patients discontinue fish oil before surgery or in any situation with bleeding risk [62]. Bays and Harris [63, 64] believe that these risks are strictly theoretical, and omega-3 fatty acids are safe to use based on a large number of RCTs that have demonstrated its safety. Nevertheless, according to Harris, whether the combined effects of glycoprotein IIb/3a inhibitors and omega-3 fatty acids are safe is yet to be determined [64].

The Omega-3 Fatty Acids for Prevention of Post-operative Atrial Fibrillation (OPERA) trial included 1516 patients undergoing cardiac surgery. Based on the results, fish oils paradoxically reduced the number of perioperative and postoperative blood transfusions. Akintoye et al. further investigated bleeding outcomes and used the cohort of the OPERA trial for the secondary analysis. Based on multiple bleeding criteria, they reported that fish oil does not increase postoperative bleeding and increased omega-3 levels are associated with a lower bleeding risk. The mechanism remains largely unknown; however, it is thought that omega-3 fatty acids also have platelet-sparing effects that have not been completely elucidated yet; thus, this is an area of future investigation [65]. Moreover, patients who are at increased risk of bleeding, such as ICU/cancer patients, were found to have reduced bleeding risk after receiving a short course of treatment with EPA and DHA 10 g/day or consuming 1.5 g/day of EPA and DHA for 52 weeks [66]. Furthermore, omega-3 PUFA were found to enhance platelet response to clopidogrel treatment after percutaneous coronary intervention by reducing ADP-induced platelet aggregation and inhibiting downstream intracellular signaling from the P2Y_12_ receptor [67].

Increased red cell redistribution width (RDW) has been reported as a capable biomarker for the prediction of cardiovascular disease, even in patients without anemia [68, 69]. A study by Takahashi et al. [70] found that EPA reduces RDW in patients with ischemic heart disease, regardless of the presence of anemia. The presumed effect is due to the preferential uptake of EPA into cell membranes, particularly red blood cell membranes, resulting in improved stability and malleability as well as less variability in the size of red blood cells. Interestingly, Takahashi et al. found a particularly stronger effect on RDW reduction in patients with diabetes with higher hsCRP levels.

The effects of PUFA are not only limited to antiplatelet effects. They could also influence the coagulation cascade by inhibiting platelet-mediated thrombin formation, which in turn could be an additional cardioprotective effect of PUFA as formation of an occlusive thrombus is prevented. Larson et al. reported that with EPA and DHA supplementation, the phospholipid membrane of platelets is altered, which inhibits the platelet membrane to form the coagulation cascade complex at the sites of vascular injury, thereby resulting in decreased rates of prothrombin and thrombin formation and ultimately clot formation [71].

## Electrophysiologic effects of omega-3 fatty acids

AF is the most common arrhythmia, which is affecting over 2–3 million patients. Patients with AF are at increased risk of stroke, adverse cardiovascular events, and re-hospitalization [72, 73]. Omega-3 PUFA have several effects on the cardiac electrical circuit, including cardiac membrane stabilization due to inactivation of the fast sodium channels and L-type calcium channels, which are responsible for depolarization resulting in a prolonged refectory period and less automaticity, which could in turn protect against fatal arrhythmias [74–76]. Evidence of omega-3 PUFA’s ability to limit atrial remodeling, which is known to predispose patients to developing AF, is available; however, the outcomes are inconsistent [77]. Nonetheless, omega-3 PUFA play a role in reversing or inhibiting the pathophysiologic processes involved in AF, such as oxidative damage/stress, inflammation, and endothelial dysfunction. Therefore, omega-3 PUFA have gained much interest as a potential intervention to prevent or treat AF [78]. The area of interest surrounding omega-3 PUFA in most RCT involves the prevention of AF in postoperative cardiac surgery patients after they spontaneously converted into sinus rhythm or maintained sinus rhythm after cardioversion.

### Maintenance of sinus rhythm after cardioversion

The Randomised Trial to Assess Efficacy of PUFA for the Maintenance of Sinus Rhythm in Persistent Atrial Fibrillation (FORWARD) trial is a randomized, double-blind, multicenter, placebo-controlled trial performed by Macchia et al. involving 586 patients in the outpatient setting, and investigated whether omega-3 PUFA 1 g/day compared to placebo (olive oil capsule) could prevent AF recurrence in patients with prior, confirmed, symptomatic paroxysmal AF requiring cardioversion. The inclusion criteria were as follows: ≥ 2 symptomatic episodes of AF 6 months before randomization, with the last episode occurring 3–90 days before randomization, and successful electrical or pharmacological cardioversion for persistent AF within 3–28 days prior to randomization. At 1-year follow-up, they found no difference in reduction of recurrent AF and in end points including all-cause mortality between omega 3 PUFA and placebo. Moreover, similar trials, which included the assessment of paroxysmal/persistent AF, found comparable results, even with a high dose PUFA [79–81]. However, potential confounding factors and limitations exist, which are largely attributed to the antiarrhythmic medications that the patients were receiving throughout the study period; these medications could affect the anti-arrhythmic effects of omega-3 PUFA and AF recurrence.

The length of treatment with PUFA also plays a significant role in terms of patient outcomes. Positive results were observed when pretreatment with PUFA was started 4 weeks before cardioversion [82, 83]. A meta-analysis including 749 patients found that omega-3 PUFA have no effect on AF prevention after cardioversion (pooled odds ratio (OR) 0.63; 95% confidence interval (CI) 0.35–1.13; p = 0.12); however, when the analysis was restricted to a specific subgroup in which PUFA were administered 4 weeks prior to cardioversion, a significant reduction in the AF recurrence was found (OR 0.39, 95% CI 0.25–0.61; p < 0.0001) [84]. The time course between effectiveness of therapy and initiation of treatment could be associated with the time required for PUFA to insert into the cell membranes and exert their action [85]. However, the patients in the studies included in the meta-analysis were treated with anti-arrhythmic drugs, which possibly confounded the results.

### Postoperative atrial fibrillation

Postoperative AF (POAF) is a common complication after cardiac surgery, with a reported incidence of approximately 30% after coronary artery bypass procedures [86]. Calo [87] and Heidt [88] showed a statistically significant decrease in the incidence of POAF in patients receiving PUFA compared to placebo. However, the FISH trial [89] reported similar rates of POAF between the treatment and placebo groups despite receiving a minimum of 6 g of fish oil and a longer treatment duration (2 weeks after surgery) than that in the studies by Calo and Heidt. In addition, Farquharson performed a double-blind RCT in patients with a 3-week treatment with fish oil prior to surgery and, surprisingly, still found no difference between the placebo and intervention groups despite the pre-surgical load of PUFA [90]. These inconsistent results contributed to the conduct of the OPERA trial, which is an international, multicenter double-blind, placebo-controlled, randomized clinical trial. Briefly, patients were randomly allocated to receive fish oil (EPA 465 mg and DHA 375 mg), with a loading dose of 10 g over 3–5 days (or 8 g over 2 days), or placebo until postoperative day 10 or discharge from the hospital, whichever came first. The primary end point was POAF occurrence for > 30 s, which occurred in 30.7% of the patients in the PUFA group and 30% in the placebo group. The secondary end points also did not reach statistical significance, which included POAF lasting for > 1 h, symptomatic AF, or AF requiring cardioversion [91]. The OPERA trial concluded that perioperative treatment with omega-3 fatty acids compared to placebo does not reduce the risk of POAF.

The type of cardiac surgery may also have a role; valve surgery (mitral) is associated with higher rates of AF occurrence compared to coronary artery bypass graft (CABG). A meta-analysis reported that preoperative PUFA have increased efficacy in patients undergoing CABG compared to those undergoing open heart valve surgery. However, this study did not perform an analysis of potential confounders, including anti-arrhythmic or pertinent cardiovascular drug use and dietary habits (amount of fish intake) [92].

The serum and phospholipid membrane levels of omega-3 also have important implications. Skuladottir [93], who examined 125 patients undergoing CABG, measured n-3 PUFA and n-6 PUFA in plasma phospholipids pre- and postoperatively. They reported that POAF is significantly increased in patients with elevated DHA levels. This finding was further supported by the following two studies: Viviani et al. evaluated 40 idiopathic atrial flutter/AF patients and 53 healthy control subjects and reported that patients with atrial flutter/AF have a higher percentage of n-3 PUFA in the erythrocyte membranes than those in the control group [94], and Tomita et al. evaluated three groups of patients using fish oil and assessed for AF development; their study, which included 36 healthy patients, 46 patients with ischemic heart disease, and 110 patients with AF, found that serum concentrations of n-3 PUFA are higher in patients with AF. Moreover, patients with AF alone had higher levels of n-3 PUFA than those with ischemic heart disease and AF. Of note, they found that serum EPA levels are higher than those of DHA in patients with AF [95].

The potential mechanism(s) for the increased risk of AF has been investigated using intravenous (IV) omega-3 PUFA. Kumar et al. randomized 88 patients without structural heart disease into two groups: the intervention group (receiving IV omega-3 PUFA) and the control group (receiving normal saline). They found no change in the atrial refractory period but reported significant lengthening of the right and left atrial conduction times without changes in the function of the SA node. Slowing the atrial conduction times provides anti-fibrillatory effects by increasing the size of re-entry circuit(s) that occurs in the atrial remodeling process. They also reported that patients receiving IV omega-3 PUFA have decreased inducibility of AF, decreased conversion of AF into atrial flutter, and a shorter duration of AF and flutter compared to the control group [96].

Interestingly, the inducibility of atrial flutter was increased without causing any changes in the structural properties of the heart. These results demonstrate that IV omega-3 PUFA have pro-arrhythmic and anti-arrhythmic properties that need to be further investigated to determine the situations in which they have the most beneficial effects without causing harm to patients with their possible pro-arrhythmic effects.

## Limitations

The REDUCE-IT trial is one of the most riveting trials contributing to the excitement behind the the use of omega-3 fatty acids, especially in the field of cardiology and diabetology. Similar to all trials, the REDUCE-IT trial and studies involving omega-3 fatty acids do not come without shortcomings.

The placebo use of mineral oil attracted much attention because of its reported action to decrease the absorption of drugs (including icosapent ethyl) and increase atherogenic lipoproteins and C-reactive protein (CRP). Triglycerides, LDL, apolipoprotein B, and CRP all increased by 2.2%, 10.9%, 7.8%, and 32.3%, respectively [97]. Similar mineral oil effects were described in the ANCHOR and MARINE studies [98, 99], although the ANCHOR trial authors reported that mineral oil is minimally absorbed and chemically inactive and the doses of mineral oil used in the studies were inadequate to interfere with absorption of fat-soluble substances.

The risk of bleeding cannot go unnoticed; in as early as 1978 [57], the risk of bleeding was documented in the Greenland Eskimo population due to the high number of EPA that exists within their cell membranes. In the REDUCE-IT trial, serious bleeding events occurred more in patients in the EPA group than in the placebo group, although serious central nervous system or gastrointestinal bleeding was not significantly higher than in the placebo group.

AF or flutter-related hospitalizations were significantly higher in the EPA group than in the placebo group in the REDUCE-IT study. Conversely, studies [82, 83] have reported an improvement of maintaining sinus rhythm with EPA after cardioversion, with better results seen in patients treated with longer durations of therapy.

The dosage of omega-3 fatty acids should be paid close attention. Many of the prior omega-3 fatty acid studies that found no cardiovascular benefit used doses ~ 1 g/day compared to the 4 grams used in the REDUCE-IT trial. Even with higher doses, results have been inconsistent. Poreba et al. [100] reported the use of 2 g/day of EPA and DHA did not improve coagulation, metabolic, or inflammatory parameters in patients with well-controlled diabetes and atherosclerotic disease. The discrepancy in results is puzzling, which further exemplifies the need for more randomized clinical trials.

Moving forward, the risk of bleeding, using mineral oil, rates of AF, dosages and durations of omega-3 fatty acids (high dose versus low dose) should be taken into consideration when designing future and ongoing randomized controlled trials. The STRENGTH trial [49] and EVAPORATE trial [101] are studies that will further evaluate the efficacy of high-dose, pure EPA in patients with cardiovascular disease, and their results are highly anticipated.

## Conclusion

In this study, we presented an overview of the known physiology of omega-3 fatty acids and demonstrated their ability to reduce triglyceride levels, atherogenesis, and platelet aggregation with an associated increase in the electrical conduction intervals within the atria. These mechanisms could be extrapolated from the results of the REDUCE-IT trial, which showed a statistically significant reduction in the composite of cardiovascular death, nonfatal MI, nonfatal stroke, coronary revascularization, and unstable angina. However, increased rates of AF were noted, with a trend towards increased bleeding rates. Given the known mechanism of omega-3 fatty acids and the purified derivative EPA ethyl ester, increased interest into their clinical applicability is possible with the data available to support its use.

## Data Availability

Data sharing is not applicable to this article as no datasets were generated or analyzed.

## Abbreviations

ACS: acute coronary syndrome
ALA: a-linoleic acid
EPA: eicosapentaenoic acid
CABG: coronary artery bypass graft
CAD: coronary artery disease
CI: confidence interval
COX: cyclooxygenase
CRP: C-reactive protein
DHA: docosahexaenoic acid
hsCRP: high-sensitivity C-reactive protein
LDL-C: low-density lipoprotein cholesterol
MI: myocardial infarction
NNT: number needed to treat
OCT: optical coherence tomography
OR: odds ratio
POAF: postoperative AF
PPAR: peroxisome proliferator-activated receptor
PUFA: polyunsaturated fatty acids
